# Novel therapeutic strategies for degenerative disc disease: Review of cell biology and intervertebral disc cell therapy

**DOI:** 10.1177/2050312118761674

**Published:** 2018-03-12

**Authors:** Joseph Fernandez-Moure, Caitlyn A Moore, Keemberly Kim, Azim Karim, Kevin Smith, Zonia Barbosa, Jeffrey Van Eps, Pranela Rameshwar, Bradley Weiner

**Affiliations:** 1 Department of Surgery, Houston Methodist Hospital, Houston, TX, USA; 2 Department of Regenerative and Biomimetic Medicine, Houston Methodist Research Institute, Houston, TX, USA; 3 Department of Medicine, Rutgers New Jersey Medical School, Newark, NJ, USA; 4 Texas A&M College of Medicine, Bryan, TX, USA; 5 Department of Orthopedic Surgery, Houston Methodist Hospital, Houston, TX, USA

**Keywords:** Stem cells, disc, intervertebral disc degeneration, disc cells

## Abstract

Intervertebral disc degeneration is a disease of the discs connecting adjoining vertebrae in which structural damage leads to loss of disc integrity. Degeneration of the disc can be a normal process of ageing, but can also be precipitated by other factors. Literature has made substantial progress in understanding the biological basis of intervertebral disc, which is reviewed here. Current medical and surgical management strategies have shortcomings that do not lend promise to be effective solutions in the coming years. With advances in understanding the cell biology and characteristics of the intervertebral disc at the molecular and cellular level that have been made, alternative strategies for addressing disc pathology can be discovered. A brief overview of the anatomic, cellular, and molecular structure of the intervertebral disc is provided as well as cellular and molecular pathophysiology surrounding intervertebral disc degeneration. Potential therapeutic strategies involving stem cell, protein, and genetic therapy for intervertebral disc degeneration are further discussed.

## Introduction

Chronic low-back pain is a leading cause of disability and is a major public health burden in the United States.^1^ According to a recent Institute of Medicine report, the financial burden is estimated to be at least US$560–US$635 billion annually, which equates to US$2000.00 per capita.^2^ In addition, productivity loss estimates stemming from missed workdays range between US$297 and US$336 billion. It has also been estimated that two-thirds of adults are afflicted with low-back pain at some point in their lives, and symptoms of lower back pain are a leading cause of visits to a physician.^3^ The costs of unrelieved back pain has been linked to prolonged hospital stays and increased outpatient visits. Herein, we will discuss the degenerative disc pathology, current strategies to intervene on degenerative disc disease, and future perspectives on treatment.

### Degenerative disc disease

Intervertebral disc degeneration, or degenerative disc disease, is a major cause of lower back pain that typically occurs with age. In this process, the intervertebral discs (IVDs) undergo changes that affect their ability to act as shock absorbers.^4^ Due to undetermined reasons, the discs slowly lose their normal protein and water content and become replaced with fibrocartilage.^4^ As a result, the disc morphology changes and ultimately leads to a decrease in disc height, making the spine less stable and causing more pressure on the posterior elements of the spine. Spinal stenosis ensues as the facet joint and ligamentum flavum undergo hypertrophic changes that lead to narrowing of the spinal canal and lateral recesses.^5^

In the early stages of the disease, treatment options such as physical therapy, analgesics, and anti-inflammatories are aimed at addressing the symptoms. In more advanced cases, epidural steroid injections can be utilized to address radicular symptoms and ultimately surgical treatment, including fusions, may be undertaken to help alleviate symptoms. Spinal fusion is surgery to permanently join together two or more bones in the spine so there is no movement between them. The surgery involves using a graft such as autologous iliac crest bone graft to join the bones together permanently. Methods of achieving this include using pieces of bone graft material being placed over the back part of the spine or between the vertebrae and using cages that are packed with bone graft material placed between the vertebrae. Spinal fusions may relieve much of the pain associated with degenerative discs, but the mechanics of the spine are compromised at levels above and below the fusion and may cause more stress during spine motion. Other processes such as adjacent segment disease may become problematic. Clinical and basic science research continues to attempt to restore the normal cellular environment of IVDs to help prevent degeneration.

## IVD cell biology

**Figure 1.**
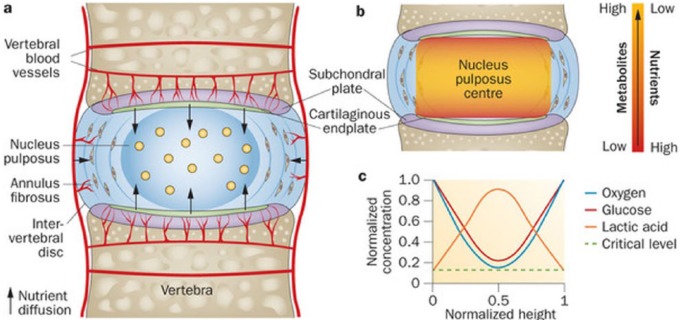
Anatomical configuration of human intervertebral disc from a sagittal perspective (a) with schematic demonstrating concentration gradient within actual disc in sagittal plane (b) and normalized concentration levels of specific metabolites demonstrating spatial relationship (c). Reproduced from Huang et al.^7^ with permission.

The human IVD consists of three disparate entities: a central nucleus pulposus (NP), outer or peripheral annulus fibrosus (AF), and hyaline cartilage endplates (CEPs) that separate vertebral bodies. AF consists primarily of type I collagen arranged in bundles of concentric rings termed lamellae. The NP is a more random arrangement of type II collagen and proteoglycans interwoven in a solid mucus, viscous material.^6^ The IVD is an avascular structure that relies on diffusion of nutrients and oxygen via a concentration gradient from the adjacent endplate (Figure 1). Major constituents of the IVD are glycosaminoglycans, aggrecans, and collagen. Cellularity tends to decline towards the NP to a low constant value. Cell density estimates of the entire disc have been reported to be 60,000/mm^3^.^8^ NP cells have been shown to survive in low oxygen environment and primarily rely on glycolysis for metabolism.^9^ In addition, mesenchymal stem cell markers have been identified in both NP and AF.^10^

AF cells have been characterized as elongated fibroblasts with extended cytoplasmic processes of mesenchymal origin.^11^ They have the capacity to synthesize proteoglycans and collagen. A unique feature of AF cells is that they can be a source of pluripotent stem cells with potential to differentiate into adipocytes, chondrocytes, neurones, osteoblasts, and endothelial cells.^12^ With ageing, AF cells become more rounded and chondrocytic compared to the typical spindle appearance and develop multiple cytoplasmic processes extending extracellularly.^13^

An extraction technique of the AF following discectomy that has been described involves dissecting out the outer material to avoid contamination of adjacent blood vessels and nerves. Collagenase is then applied to remove the aqueous phase, which is then centrifuged at 500*g* for 10 min. The cell pellet is placed in an erythrocyte lysis solution, re-centrifuged, then re-suspended in culture media containing foetal bovine serum/antibiotic and the cells stored in a culture dish within an incubator that require standard cell culture care and sequential passaging.^12^

The NP contains two cell types: notochordal cells and chondrocytes.^14,15^ Notochordal cells are larger, ranging from 25 to 85 µm in diameter compared to 17–23 µm in chondrocytes. The chondrocytes contain more mitochondria, rough endoplasmic reticulum packed with glycogen, and cytoplasmic filaments compared to notochordal cells.^15^ Protein expression levels of cytoplasmic and trans-membrane proteins such as CD44s, galectin-3, vimentin, cytokeratins 8 and 19, and collagen type IIA have been demonstrated in notochord cells.^16^ The adaptability of notochordal cells in a hypoxic environment has been demonstrated through up-regulation of hypoxia inducible factors and vascular endothelial growth factor (VEGF) compared to AF cells.^17^ The role of notochordal cells in development is still not fully understood. There is evidence supporting a role in directly creating NP and then differentiating into chondrocytes. They may also simply orchestrate cell movements and proteoglycan synthesis of surrounding cells followed by senescence.^18^ Notochordal cells are more able to make proteoglycans than the other cells, which may make them responsible for maintaining the gelatinous consistency.^19^ Interestingly, notochordal cells may no longer present by the age of 10 years in humans, which possibly correlates with the onset of disc degeneration.^15^

The cells from the NP can be separated using fluorescence-activated cell sorting (FACS) or Ficoll-Hypaque density gradient centrifugation.^20^ Size difference allows for separation based on side-scatter and forward-scatter parameters. Once separated, studies can be conducted to determine differences between AF and NP cells such as protein and RNA characterization.

The molecular biology of the IVD is a complex interplay of growth factors, genes, and proteases with multiple areas of coordination to maintain homeostatic mechanisms. Multiple reports have linked fibroblast growth factor (FGF) in herniated and degenerated IVD in humans.^21,22^ Insulin-like growth factor-I (IGF-1) and platelet-derived growth factor (PDGF) has been overexpressed in herniated human IVDs in addition to being expressed in rabbit and bovine IVDs.^23^ Transforming growth factor beta (TGF-β) regulates synthesis of collagen and proteoglycans, but also has role in IVD anaerobic metabolism. In canine IVDs, TGF-β administration led to increased proliferative responses as measured by 3H-thymidine incorporation and 35S-sulphate incorporation into proteoglycan compared to FGF.^24^ Using RT-PCR techniques in herniated human IVD, TGF-β demonstrated a role in maintenance and degenerating processes of the IVD.^25^ Loss of TGF-β signalling in growth plate chondrocytes and inner AF cells has been shown to be a precursor to loss of matrix tissue and endplate cartilage cells and abnormal growth plate cartilage morphology.^26^ Bone morphogenetic protein (BMP) is also a multi-functional growth factor that has shown to play a pivotal role in regulating events in the IVD, such as increasing synthesis of proteoglycans, regulating mRNA expression of type II collagen, and serving as a mitotic agent.^23^ In vitro application of BMP-2 stimulated rat IVD cell proliferation and disc extracellular molecules. It also unregulated mRNA expression of many chondrogenic components such as type II collagen and aggrecan in human disc cells.^27^ These findings represent the difficulty of manipulating a complex biological system with one signal or growth factor. A more biomimetic, biologically similar, approach is required to recapitulate the environment you are trying to recreate. Unfortunately, the regulatory hurdles to bring such a growth factor cocktail to the clinic are great, and thus, attention has been turned towards other approaches to supplying the injured tissues with growth factors.

Platelet-rich plasma (PRP) has been an alternative strategy in IVD regeneration with documented success due to its multiple growth factors.^28,29^ Activated platelets in PRP release over 70 bioactive factors, such as BMP, connective tissue growth factor, FGF, and TGF-β3. However, single growth factor injection may have limitations, as it seems that no single growth factor is potent enough to reverse the degenerative cascade. Delivery of PRP via gelatine-based hydrogel microspheres into the NP of degenerated rabbit discs suppressed progression of disease significantly.^30^ Further analysis of therapeutic effects of this PRP model indicated mRNA expression levels of type II collagen were higher, along with greater disc height and preservation of water content. In addition, a rat model of degenerated IVD with addition of PRP appeared to be protective based on magnetic resonance imaging (MRI) findings of retained morphological features, reduced inflammatory cells, and increased water content. A similar rabbit model of degenerated IVD with PRP injections also led to significant restoration of disc height and recruitment of chondrocyte-like cells.^31^ Experimental protocols in human IVD remains to be a source of further investigation.

### Disc cells and ageing

With ageing and degeneration, disc cells experience several biologic changes. Changes in cell type in the NP begins in childhood with the disappearance of notochordal cells in the NP. This disappearance is correlated with the transformation of a fluid-like NP to a more cartilaginous-based structure. Mixed results have been reported in regard to cell density, with animal models supporting a decrease in cellularity^32^ while some investigators reported that cell densities increased in human disc cells over time.^8,33^ However, no distinction of cell viability was ascertained in those reports.

Factors influencing IVD cell death are nutrient supply, mechanical stresses, and temporal decline in cell viability due to ageing alone. Since the thickness of the endplates diminishes with age, nutrition is impaired to the cells and affects viability.^34^ Experiments in a mouse IVD model revealed a linear correlation of IVD cell apoptosis as static compression loads on the endplates were increased.^35^ As for cell proliferation, the consensus among several reports is that cell proliferation increases with age in human IVD and in degenerative discs.^36^ The exact mechanism and reason for this biological behaviour remain uncertain. Cultured IVD cells have shown proliferation by growth factors such as TGF-β1, BMP-2, and osteogenic protein-1.^37^ IVD cell phenotype changes over time have also been extensively reported in the literature. Briefly, it was characterized as having a reduced ability to synthesize appropriate matrix constituents, catabolic metabolism, pro-inflammatory state, and reduced growth factor secretion.^38,39^ Finally, IVD cell senescence plays a role in ageing and degeneration. Human-herniated discs have demonstrated higher expression of senescence-associated beta-galactosidase (SA beta-gal) compared to controls and higher concentration was noted in the NP.^40^ Immunohistochemical localization studies of SA beta-gal further supported the role of increased IVD cell senescence in human degenerative disc model.^41^

## IVD therapy

To prevent disc degeneration, the abnormal conditions of the decreased viable cell population and the altered cell phenotype are a target for correction. Hence, by better understanding the biological processes underpinning these phenotypic changes, cellular therapies can be more effectively designed to target specific dysfunctions. Support for this comes from growth factor infusion experiments where injections of TGF-β in degenerate mouse IVD led to increased cellularity and disc height.^42^ Rabbit IVDs exposed to injections of BMP-7 also led to restoration of disc height and increased proteoglycan content.^43^ Alternatively, NP cells injected in degenerative disc disease rabbit models showed reduction in the decline of disc height, increased T2-weighted signal intensity, and higher glycosaminoglycan (GAG) content.^44^ Although in vivo results in animal models have been encouraging, limitations to this approach are that IVD degeneration is a complex interplay of multiple growth factors, genes, and epigenetic processes that single factor administration may not be able to completely restore. In addition, temporal expression patterns have not been established for many growth factors in human discs.

Several recent studies have revealed the presence of cell niches and progenitor cells in IVD tissues.^10,45–48^ The presence of these cells indicates that natural repair mechanisms exist in IVDs. However, during ageing and regeneration, progenitors fail to repopulate and regenerate dysfunctional IVD tissue. In particular, NP progenitor cells (NPPCs) may represent a key cell type responsible for regenerative capacity of IVD NP as NPPCs may contribute to lifelong tissue homeostasis in health and their exhaustion may be responsible for degeneration in disease and ageing. Sakai et al.^49^ first identified NPPCs in the Tie2+ and GD2+ subpopulation of NP cells in mice and human IVDs. These data were later corroborated in a bovine coccygeal model.^50^ Tie2 is a receptor tyrosine kinase reported to be expressed in haematopoietic and neural stem cells;^51–53^ GD2 is a plasma membrane marker recently identified as a marker for bone marrow and umbilical cord mesenchymal stem cells (MSCs).^54,55^ Furthermore, Sakai et al. indicate that Tie2 ligand angiopoietin-1 maintains the NPPCs and protects NP cells from apoptosis. Strategies to maintain Tie2+ NPPCs require further investigation.

In addition, studies have demonstrated that both non-degenerative and degenerated IVDs contain AF-specific progenitor cells.^12,45,56^ Moreover, progenitor cells have also been identified within the CEP.^47^ AF and CEP progenitor cells show similarities to bone marrow–derived MSCs. All things considered, isolating distinct progenitor cell populations may provide a more effective, tissue-specific cell source for cell-based therapies aimed at IVD degeneration. Also, incorporation of IVD tissue-specific progenitors into biomimetic scaffolds would significantly impact the regeneration potential and efficacy of tissue-engineered IVD constructs. Improved clinical outcomes are promising through either modality. Nevertheless, further analyses are needed to develop reliable methods with which to isolate, maintain, and expand these progenitor cells for use in clinical therapeutics.

Restoration of proteoglycan quantity and quality in degenerated IVD is also an important component to regeneration of IVD. SOX9 has been found to be an important transcription factor in the process of type II collagen synthesis and is a promising gene target for IVD regeneration through gene transfer or gene therapy. IVD cells transfected with adenovirus-mediated SOX9 demonstrated increased proliferation and synthesis of proteoglycans.^57^ An ex vivo gene transfer model has also been established using cultured human IVD cells transfected with adenoviral vectors carrying inhibitor to interleukin-1 (IL-1), an important cytokine in the inflammatory cascade, in degenerate IVD.^58^

### Tissue engineering and biomaterials

Tissue engineering aims to recapitulate the structure and physical properties of the tissue while facilitating regeneration of the tissue through the incorporation of relevant stem cell types. This approach differs from those mentioned previously as it focuses more on tissue regeneration than recovery. Cell-loaded biomaterial constructs have become a subject of investigation to address degenerated IVD structure and function. In theory, scaffolds provide more effective regenerative cell therapy by providing a three-dimensional (3D) microenvironment to help retain cell morphology and provide mechanical stability. In IVD tissue engineering, biomaterial scaffolds must be able to withstand physiological forces of the spine and able to cultivate stem cells in such a way that they are able to synthesize tissue de novo. Choosing the correct type of stem cells is imperative for obtaining favourable results in regenerative medicine. Bone marrow and adipose-derived MSCs are most frequently utilized for this application.^59^ However, studies are currently being conducted using MSCs from umbilical cord Wharton’s jelly.^60^ MSC-transplanted discs have shown histologically preserved structure, including mitigated NP cell depletion and decreased disorientation of annular structure compared to untreated controls.^61^ In addition, hydrogels serve as a retainer of the MSCs, preventing their leakage into the intervertebral space, which causes osteophyte formation.^62^

Injectable collagen hydrogels have been proposed as a potential biomaterial construct for NP tissue to minimize damage to the surrounding AF. Particularly, collagen hydrogels containing MSCs have proven successful in animal studies of engineered IVDs. Atelocollagen, a collagen gel, has been studied in vitro and provides a biocompatible environment that augmented NP cell function.^63,64^ In vivo implantation of AF cells seeded in atelocollagen scaffolds in a rabbit model prevented progression of IVD space narrowing and had viability and proliferative activity.^65^ A recent study in which MSCs in collagen hydrogel were injected into the NP of damaged rabbit IVDs showed significant differences in disc height after 8 weeks when compared to discs injected with cell-free hydrogel and untreated discs.^4^ Several studies have shown that the use of collagen-mixed mediums in a gel-like form has the consistency of native NP, which along with exposure to low levels of oxygen enhances MSC differentiation towards NP cell type.^4,66^ This is consistent with other studies, indicating that differentiation of MSCs depends largely on the local microenvironment.^4,67–69^

MSCs bi-directionally communicate with NP cells during co-culture, suggesting that implanted MSCs may influence NP cell function through secretion of bioactive factors. Recent evidence shows that MSCs possess anti-inflammatory and anti-catabolic properties that can be used to reduce elevated inflammatory cytokine levels detected in the degenerated IVD microenvironment. Modulation of the inflammatory niche would produce a healthier, non-degenerative phenotype in native NP cells. MSC and NP cell co-culture studies have found a beneficial paracrine relationship in that MSCs deliver miRNA-21 to NP cells via exosomes to inhibit NP cell apoptosis and reduce IVD degeneration.^70^ To promote disc formation by MSCs, culturing in notochord cell–conditioned media (NCCM) has been reported to increase secretion of GAG and type III collagen, resembling function of NP cells in early IVD development.^71^ Studies have shown that injectable decellularized extracellular matrix (ECM) has been found to maintain structural and compositional features of native tissue by increasing GAG and ECM component production and promoting NP cell and MSC adaptation.^72^ Such studies indicate that paracrine signalling may be responsible for the observed effects.

Other biomaterials used for tissue engineering scaffolds include fibrin, alginate, silk, gelatine, PLGA, small intestine submucosa, hyaluronan gel, and genipin cross-linked chitosan.^69,73,74^ Recently, such materials have been used in composite to create biphasic scaffolds. Biphasic scaffolds are an attempt to engineer whole IVD by recapitulating the unique structures and functions of both NP and AF.^75,76^ For instance, a biphasic scaffold was fabricated in which silk proteins are used for the AF and fibrin and hyaluronic acid (HA) gels for the NP.^77^ AF cells and chondrocytes were seeded onto the scaffold and effectively stimulated both AF and NP tissues and were effective in the formation of the total IVD in vitro. In another study, researchers developed a novel integrated biphasic IVD comprised freeze-dried and cross-linked porcine bone matrix gelatine for the AF and porcine acellular cartilage for ECM for the NP.^78^ AF and NP were seeded with porcine cells native to respective fractions. IVD-like tissue was observed in this model after 6 weeks of implantation in nude mice.

**Figure 2.**
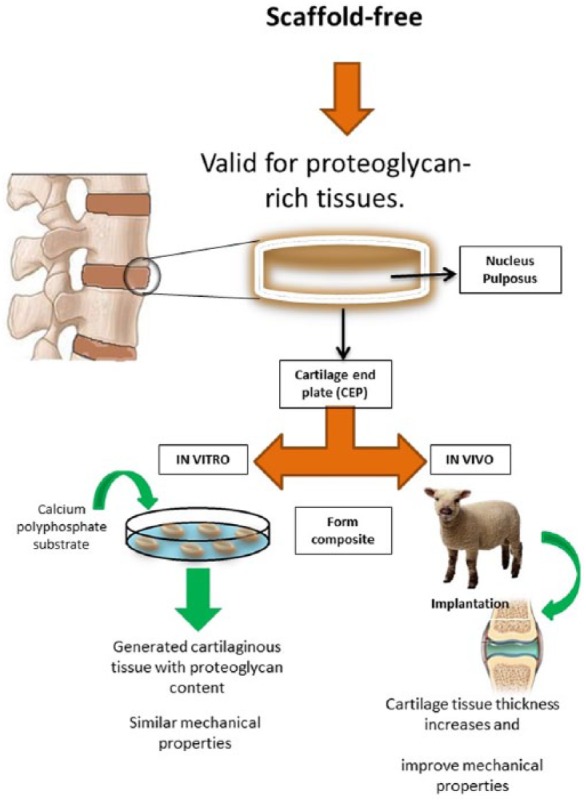
Experimental design summary and strategy for testing scaffold-free tissue engineering and assessing regenerative potential. The scaffold-free approach involves culturing cells to produce ECM that will ultimately serve as the IVD implant. In vitro studies assess generated cartilaginous tissue while in vitro studies assess increases in cartilage tissue thickness.

Another novel approach to scaffold development that is emerging is based on the inherent ability of cells to form their own matrix, much like that of the destination tissue.^74,79^ This approach involves culturing cells to produce ECM that will ultimately serve as the IVD implant (Figure 2). Properties of ‘scaffold-free tissue’ will depend on the recipient tissue biomechanical properties and the tissue from which the cells were obtained.^80^ Tissues that require high mechanical strength such as long bones of the abdominal wall may not be suitable for this approach. However, within the context of IVD tissue engineering, this approach may be a useful for formation of composite tissues such as NP or cartilage.^81^

**Figure 3.**
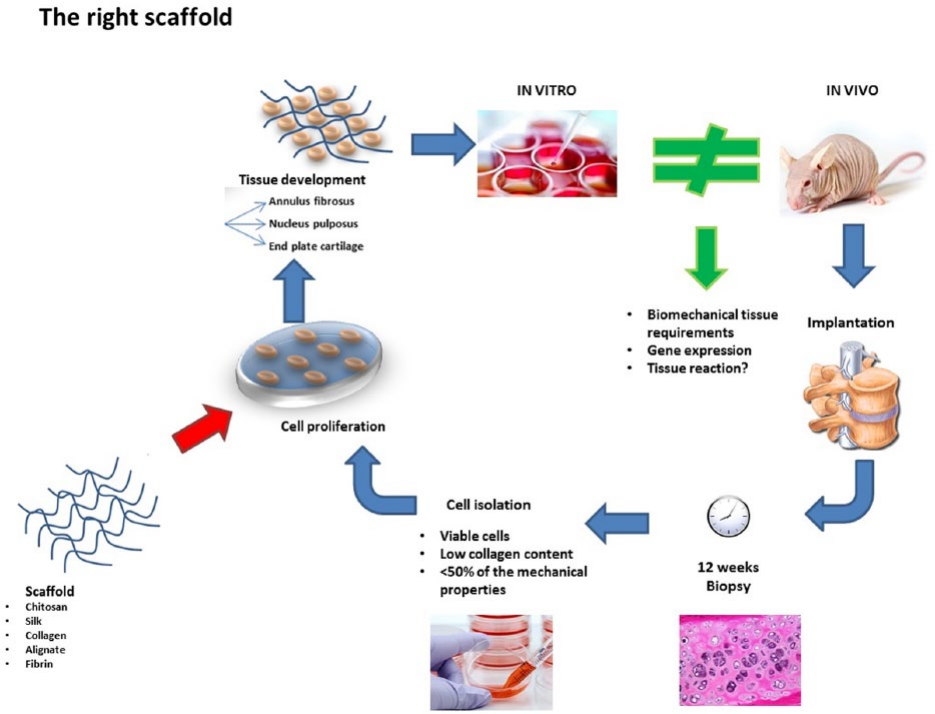
In vitro and in vivo investigation to understand scaffold-cell interactions, cell orientation and the cellular microenvironment and identify the optimal scaffold and conditions that satisfy the three biological components of the IVD.

Researchers perform both in vitro and in vivo investigations to understand scaffold–cell interactions, cell orientation and the cellular microenvironment leading to a recipient tissue reaction (Figure 3).^82–86^ By better understanding the tissue reaction in vitro, materials can be tuned to deliver specific topographical and biochemical signals to implanted cells and native tissue. In vivo studies are needed to identify the optimal scaffold and conditions that satisfy the three biological components of the IVD.^81^ Although there are several promising avenues emerging for the regeneration of IVD, many challenges arise when translating in vitro systems into in vivo models, such as the small animal models discussed. Engineered IVD constructs must be able to withstand physiological loading to be effective. Although this property can be predicted in the laboratory, in vitro measurements are not always indicative of in vivo outcomes. Furthermore, cells within scaffolds in vitro are under controlled conditions. Once implanted in vivo, the microenvironment becomes much more complex, impacting viability, differentiation, and other important cell functions.

### Translation to clinic

Clinical trials of human IVD regeneration using MSCs have had promising initial results in a limited number of trials. Autologous bone marrow MSCs injected into the NP of 10 patients with chronic back pain resulted in improvement of clinical symptoms in 1 year with no adverse events but also with no changes on MRI.^87^ Similar clinical resolution was noted after 2-year follow-up in two patients that had autologous MSCs implanted percutaneously in the degenerated IVD.^88^ Alternatively, re-implantation of isolated IVD disc cells that are then stimulated in conditioned media from damaged IVD back into the same degenerated areas has been attempted with the intent to increase quantity of viable cells and restore ability to synthesize extracellular components. In a canine model, the EuroDISC trial demonstrated at 2-year follow-up disc cell viability, proliferative capacity, ECM synthetic ability, proteoglycan content, and reduction in pain scores.^89^ Large-scale, randomized with placebo clinical trials remain areas of further investigation.

In light of the aforementioned clinical successes, many challenges stem from the translation of proposed IVD technologies to clinic. Since the differentiation of MSCs depends largely on the microenvironment in which they are seeded, difficulties in effective MSC differentiation into NP cells have emerged.^67^ Another complicating factor is the normal NP is relatively acellular and avascular, with cellular densities shown to vary with age.^61,66^ As a result, cells in the NP are dependent on nutrient diffusion from the endplates in a largely ineffective and poorly efficient process.^8^ Understanding these changes in the microenvironment of the IVD will help guide subsequent treatment options. Concentrations and cell types present at various ages will assist in deciding which interventions will be most fruitful to investigate. Also, mode of delivery plays an important factor in translation of tissue-engineered constructs from animal model to clinic, as delivery routes may differ between species as a result of size. Similarly, animal models are advantageous for testing and optimization, but are not entirely representative of physiological loading of the human spine.

As with many innovative approaches to an age-old pathology, IVD therapy has been slow to achieve clinical translation and efficacy. Because of the specificity of the microenvironment of the NP, it is vital that cells conditioned to the NP environment be used in order to achieve the best therapeutic effect. In bone tissue engineering, it has been demonstrated that use of the MSCs isolated from the tissue that you intend to regenerate can have a greater potential for target tissue regeneration.^90^ We believe the use of native NP cells for re-implantation will have the highest chance of tolerating the unique environment of the NP and thus, strategies to address this should be undertaken. One method, previously mentioned is the use of biomimetic scaffolds, used to prime the implanted cells to the harsh environment of the NP. As our knowledge and use of tunable biomaterials advances, so does their utility in complex surgical scenarios. In conclusion, a multi-disciplinary approach combining both NP specific cells in a biomimetic scaffold tuned to promote regeneration in the harsh disc environment will likely find success (Table 1).

**Table 1.** Summary of therapeutic strategies discussed in the review.

Study(s)	Results and mechanism	References
TGF-β injections in degenerate mouse IVD	Results: increased cellularity and disc height Mechanism: TGF-β regulates synthesis of collagen and proteoglycans and has a role in IVD anaerobic metabolism	Walsh et al.^42^
Rabbit IVDs exposed to injections of BMP-7	Results: restoration of disc height and increased proteoglycan content Mechanism: bone morphogenetic protein (BMP) is a multi-functional growth factor. Increases synthesis of proteoglycans, regulates mRNA expression of type II collagen and serves as a mitotic agent.	Le Maitre et al.^23^ and Masuda et al.^43^
NP cells injected in degenerative disc disease rabbit models	Results: reduction in the decline of disc height, increased T2-weighted signal intensity, and higher glycosaminoglycan (GAG) content Mechanism: increase NP cellularity	Feng et al.^44^
(a) PRP delivery via gelatine-based hydrogel microspheres into the NP of degenerated rabbit discs; (b) addition of PRP in degenerated IVD of rat model; (c) PRP injections in degenerated IVD of rabbit model	Results: (a) significant suppressed progression of disease; (b) MRI findings of retained morphological features, reduced inflammatory cells and increased water content; (c) significant restoration of disc height and recruitment of chondrocyte-like cells Mechanism: activated platelets in PRP release over 70 bioactive factors	Nagae et al.^30^ and Obata et al.^31^
IVD cells transfected with adenovirus-mediated SOX9	Results: increased proliferation and synthesis of proteoglycans Mechanism: SOX9 has been found to be an important transcription factor in the process of type II collagen synthesis	Paul et al.^57^
In vivo implantation of AF cells seeded in atelocollagen scaffolds in rabbit model	Results: prevented progression of IVD space narrowing. AF cells had viability and proliferative activity Mechanism: atelocollagen provides a biocompatible environment that augments NP cell function	Sato et al.^63^, Sakai et al.^64^ and Sato et al.^65^
MSCs in collagen hydrogel were injected into the NP of damaged rabbit IVDs	Results: significant differences in disc height after 8 weeks compared to discs injected with cell-free hydrogel and untreated discs Mechanism: the use of collagen-mixed mediums in a gel-like form similar to the consistency of native NP and exposure to low levels of oxygen enhances MSC differentiation towards NP cell type	Subhan et al.^4^ and Kumar et al.^66^
(a) Biphasic scaffold was fabricated in which silk proteins were used for AF and fibrin and hyaluronic acid (HA) gels for NP. AF cells and chondrocytes were seeded onto scaffold; (b) implantation of integrated biphasic IVD comprised freeze-dried and cross-linked porcine bone matrix gelatine for the AF and porcine acellular cartilage for ECM for the NP in nude mice. AF and NP were seeded with porcine cells native to respective fractions.	Results: (a) AF and NP tissues stimulated and effective formation of total IVD in vitro; (b) IVD-like tissue was observed in this model after 6 weeks of implantation Mechanism: biphasic scaffolds recapitulate the unique structures and functions of both NP and AF	Choy and Chan^75^, Elsaadany et al.^76^, Park et al.^77^ and Xu et al.^78^
(a) Autologous bone marrow MSCs injected into the NP of 10 patients with chronic back pain; (b) two patients had autologous MSC’s implanted percutaneously in degenerated IVD	Results: (a) improvement of clinical symptoms in 1 year with no adverse events but no changes on MRI; (b) improvement of clinical symptoms at 2-year follow-up	Orozco et al.^87^ and Yoshikawa et al.^88^
Re-implantation of isolated IVD disc cells stimulated in conditioned media from damaged IVD back into the same degenerated areas in canine model	Results: at 2-year follow-up, there was disc cell viability, proliferative capacity, extracellular matrix synthetic ability, proteoglycan content, and reduction in pain scores	Meisel et al.^89^

TGF-β: transforming growth factor beta; IVD: intervertebral disc; NP: nucleus pulposus; PRP: platelet-rich plasma; AF: annulus fibrosus; MSC: mesenchymal stem cell.

## Conclusion

Degenerative disc disease due to IVD pathology leading to chronic low back pain remains a critical public health problem. Current medical and surgical management strategies have shortcomings that do not lend promise to be effective solutions in the coming years. With advances in understanding the cell biology and characteristics of the IVD at the molecular and cellular level that have been made, alternative strategies for addressing disc pathology can be discovered. Current IVD therapies such as mesenchymal stem cell infusion, IVD cell isolation and reconditioning, PRP infusion, and tissue engineering and biomaterial-based strategies represent areas of promise currently that are being actively investigated.
